# Postmortem Determination of Short-Term Markers of Hyperglycemia for the Purposes of Medicolegal Opinions

**DOI:** 10.3390/diagnostics10040236

**Published:** 2020-04-19

**Authors:** Karolina Nowak, Tomasz Jurek, Marcin Zawadzki

**Affiliations:** Department of Forensic Medicine, Faculty of Medicine, Wroclaw Medical University, J. Mikulicza-Radeckiego 4 Street, 50-345 Wrocław, Poland; tomasz.jurek@umed.wroc.pl (T.J.); marcin.zawadzki@umed.wroc.pl (M.Z.)

**Keywords:** short-term markers, hyperglycemia, postmortem diagnosis, forensic medicine

## Abstract

Diabetes mellitus is classified as the epidemic of the 21st century. Due to the fact that acute carbohydrate metabolism disorders usually do not indicate morphological change, postmortem diagnosis is required to perform biochemical tests. The authors decided to evaluate the usefulness of determining glucose, lactate, acetone, β-hydroxybutyric acid (BHB), and 1,5-anhydroglucitol (1,5-AG) in postmortem blood/serum, urine, and vitreous humor (VH). Biological material was collected during autopsies. The study group consisted of 50 diabetics, while the control group consisted of 50 non-diabetics, who died a sudden death, with negative test results for the presence of ethyl alcohol and were not resuscitated before death. Statistical analysis was performed using the IBM SPSS Statistics 25 software package. The most statistically significant difference between the two groups was observed for mean 1,5-AG concentration. The authors found many correlations between the concentration of the examined markers in different materials, mainly between blood/serum and VH. The most suitable short-term glycemic marker in postmortem diagnosis is 1,5-AG. Diagnosis may be supported with determinations of acetone and BHB. For medicolegal assessment, the interpretation of the biochemical test results should comprise information on circumstances of death, medical history, results of other toxicological and histopathological tests, and autopsy report.

## 1. Introduction

Diabetes mellitus is considered to be an epidemic of the 21st century. According to the data of the International Diabetes Federation (IDF), in 2019 there were approximately 463 million adults aged between 20 and 79 years living with diabetes worldwide and by 2045 this number is expected to rise to 700 million, including 68 million in Europe. It is estimated that over half (50.1%) of the patients are undiagnosed and in 2019 approximately 4.2 million people globally (aged 20–79) died of complications of the primary disease [1]. The rise of the disease is undoubtedly a challenge for clinicians, but also for forensic pathologists and toxicologists. Deaths due to acute carbohydrate metabolism disorders usually do not show morphological changes [2]. In many cases, this makes it impossible for a pathologist to make a final diagnosis exclusively on the basis of the autopsy results and standard histopathological and toxicological tests [3]. Fatal metabolic disorders, often characterized by a dynamic course, may occur at any stage of the disease [4]. This is due to the significant effect of the activity of hormones that counter-regulate insulin secretion (e.g., glucagon, cortisol, catecholamine, growth hormone) as well as insufficient activity of insulin itself [5]. Acute hyper- and hypoglycemia cause sudden death in different age groups, including children with type 1 diabetes mellitus. Therefore, a postmortem diagnosis of glycemic disorders may require the performance of biochemical tests not only to determine the cause of death in the so-called ‘undetermined autopsies’, but also to increase the detectability of the primary disease itself.

Many authors suggest postmortem diagnostics of glycemic disorders should rely on short- and long-term markers such as ketone bodies, insulin, C-peptide, 1,5-anhydroglucitol, glycated albumin, and glycated hemoglobin [6,7,8,9].

The aim of our study was to evaluate the usefulness of short-term glycemic markers: glucose, lactate, acetone, β-hydroxybutyric acid (BHB), and 1,5-anhydroglucitol (1,5-AG) in postmortem hyperglycemia diagnosis for the purposes of medicolegal assessment. In addition, we decided to check the correlation between the concentrations of each marker in individual biological matrices and evaluate the assessment of the correlation (positive and negative) between concentrations of the analyzed markers in different study groups.

## 2. Experimental

### 2.1. Study Design, Samples Collection, and Storage

Samples of peripheral blood, urine, and vitreous humor (VH) were collected during autopsies at the local forensic medicine unit and put into plastic tubes without anticoagulant. Then, they were centrifuged and the supernatant was transferred to Eppendorf tubes and frozen at −70 °C until the time of analysis. Part of the collected blood was transferred without centrifugation to Eppendorf tubes and frozen at −70 °C.

In order to determine acetone and BHB, samples of whole blood, urine, and VH were collected into glass tubes with sodium fluoride during autopsies and stored at 4 °C until the time of analysis.

The collected cases were divided into two groups: the study group, consisting of 50 subjects with ante-mortem diagnosis of diabetes; and the control group, consisting of 50 subjects who died a sudden death, with negative results of toxicological tests for the presence of ethyl alcohol (in whole blood, urine, and VH), who were not suspected of dying from glycemic disorders, and who were not resuscitated before death. Detailed data concerning the subjects, e.g., sex, age, body mass index, and time from death to autopsy/sampling, measured in days (PMI) were discussed in an earlier study [10].

Ethical Approval: All procedures performed in studies involving human participants were in accordance with the ethical standards of the institutional and/or national research committee and with the 1964 Helsinki declaration and its later amendments or comparable ethical standards. This work is part of a research grant approved by the Institute’s Ethics Committee.

### 2.2. Chemicals and Reagents

The following chemicals and reagents were used in the current study: Glucose HK Gen.3 (GLUC3; cobas^®^, Roche Diagnostics GmBH, Mannheim, Germany); Lactate Gen.2 (LACT2; cobas^®^, Roche Diagnostics GmBH, Mannheim, Germany); PreciControl ClinChem Multi 1 (cobas^®^, Roche Diagnostics GmBH, Mannheim, Germany); PreciControl ClinChem Multi 2 (cobas^®^, Roche Diagnostics GmBH, Mannheim, Germany); Multicomponent Alcohol Calibrator Kit 250–4000 µg/mL (Cerrilant, Round Rock, TX, USA); 1-propanol 99.8 ± 0.1% (CPA chem, Stara Zagora, Bulgaria); water (Chromasolv^®^ LC-MS, Sigma-Aldrich, Steinheim, Germany); acetonitrile (Chromasolv^®^ LC-MS, Sigma-Aldrich, Germany); Sodium dl-3-hydroxybutyrate (Chiron, Trondheim, Norway); Sodium dl-3-hydroxybutyrate-3,4,4,4-d_4_ (Chiron, Trondheim, Norway); diethyl ether ≥99.7%, (Sigma-Aldrich, Steinheim, Germany); Na_2_SO_4_ pure p.a. (Chempur, Piekary Slaskie, Poland); N-tert-Buthyldimethylsilyl-N-methyltrifluoroacetamide with 1% tert-Butyldimethylchlorosilane ≥95% (Sigma Aldrich, St. Luis, MO, USA); 1,5-Anhydro-d-glucitol (trc-canada, Toronto, ON, Canada); 1,5-Anhydro-d-glucitol-^13^C_6_ (trc-canada, Toronto, ON, Canada); Biochemistry control serum I (Biosystems, Barcelona, Spain); and Albumin Standard 4.0 g/dL (Pointe Scientific Inc., Canton, MI, USA).

### 2.3. Sample Preparation

#### 2.3.1. Glucose and Lactate

Due to the use of an automatic analyzer, the samples (serum, urine, VH) did not require additional processing prior to analysis after thawing and thorough mixing.

#### 2.3.2. Acetone

A 0.1 mL volume of biological material (blood, urine, VH) was placed into a 20 mL HS (headspace) glass vial with addition of 2 mL of internal standard (1-propanol, in a final concentration in sample of 0.24 mg/mL) and sealed with aluminum headspace cap with center hole. The injection volume was 1 µL.

#### 2.3.3. β-hydroxybutyric Acid

A 0.1 mL volume of biological material (blood, urine, VH) was placed into a 2 mL Eppendorf tube and spiked with 0.01 mL of sodium DL-3-hydroxybutyrate-3,4,4,4-*d*_4_ (BHB-*d*_4_, 100 µg/mL). Then, 0.25 mL of saturated Na_2_SO_4_ solution was added. Liquid–liquid extraction was carried out for 10 min using 1 mL of diethyl ether (2500× *g*). The samples were centrifuged at 12,300× *g* for 10 min. The organic phase was placed into 2 mL Eppendorf tubes and evaporated at 40 °C to dryness under a stream of nitrogen gas. The dry residues were dissolved in 0.02 mL of acetonitrile and 0.03 mL of N-tert-Butyldimethylsilyl-N-methyltrifluoroacetamide with 1% tert-Butyldimethylchlorosilane and derivatized (80 °C, 0.5 h). After cooling, the solution was transferred into glass inserts of autosampler vials. The injection volume was 2 µL.

#### 2.3.4. 1,5-anhydroglucitol

To determine 1,5-AG, we modified the method of Hess et al. [11]. A 0.05 mL of biological material (blood, serum, VH) was placed into a 2 mL Eppendorf tube with addition of 0.01 mL of internal standard (1,5-AG-^13^C_6_, 100 µg/mL). Subsequently, precipitation was carried out using acetonitrile (1:3 ratio). Samples were shaken for 10 min. (2500× *g*) and centrifuged at 12,300× *g* for 10 min. Then, the appropriate amount of supernatant was transferred into glass inserts of autosampler vials. The injection volume was 2 µL.

### 2.4. Instrumentation

#### 2.4.1. Glucose and Lactate

Glucose and lactate determinations were performed on a COBAS INTEGRA 400 plus biochemical analyzer (Roche Diagnostics Ltd., Rotkreuz, Switzerland) used for routine clinical determinations.

#### 2.4.2. Acetone

We used an Agilent 7890A GC System and a G1888 Network Headspace Sampler (Agilent Technologies, Wilmington, DE, USA). The conditions for GC were as follows: isothermal oven with temperature of 40 °C; split ratio 6:1; helium carrier gas (grade 5.0); two Flame Ionization Detectors (FID-1 and FID-2) with temperature of 300 °C and fuel flow (helium) of 30 mL/min; utility flow (air flow), 400 mL/min; and makeup flow (nitrogen), 25 mL/min. Two columns were used: DB-ALC1 30 m × 0.32 µm × 1.8 µm and DB-ALC2 30 m × 0.32 µm × 1.2 µm (J&W, Agilent, Wilmington, DE, USA); column flow rates were 3.00 mL/min in column 1 and 3.04 mL/min in column 2; front inlet was set at 150 °C, flow at 26 mL/min, septum purge flow at 5mL/min, and pressure at 89.4 kPa. The total time for one cycle was 5 min.

#### 2.4.3. β-hydroxybutyric Acid

Analyses were performed using a GC-2010 Plus chromatograph (Shimadzu, Kyoto, Japan) equipped with a split/splitless injection port (an AOC-20i auto injector and an AOC-20s auto sampler). The separation was done using a ZB-5MSi column (50 m × 0.25 mm × 0.25 μm, Phenomenex, Torrance, CA, USA). Helium (grade 6.0) was used as carrier gas (column flow, 2.21 mL/min; linear velocity, 53.9 cm/s; purge flow, 3.0 mL/min). The oven temperature gradient program was as follows: hold an initial temperature of 50 °C for 1 min, ramp to 190 °C at 25 °C/min, then ramp to 300 °C at 50 °C/min, and held for 2 min. A solvent cut time of 6 min was used. The temperature of the injection port was 250 °C. The injection mode was splitless.

The GC-2010 Plus was directly interfaced to a GCMS-TQ8040 triple quadrupole mass spectrometer (Shimadzu, Kyoto, Japan). Mass spectrometry (MS) analyses were conducted in positive electron ionization (EI) mode. The temperature of ion source was 250 °C, and the interface temperature was 300 °C. Gas argon (grade 6.0) was used as the collision-induced dissociation (CID). Determination of the investigated analytes was carried out in the multiple reaction monitoring (MRM) mode. MS running time ranged from 6.5 to 10.5 min. Total analysis time was 10.8 min. The specific transitions, optimum collision energies (CEs), event times and retention times for BHB, and internal standard (BHB-*d*_4_) are listed in Table 1.

#### 2.4.4. 1,5-anhydroglucitol

Analyses were performed using an ultra-high-performance liquid chromatograph (Nexera X2, Shimadzu, Kyoto, Japan). The separation was done using a Kinetex^®^ 2.6 µm Biphenyl 100Å LC Column 50 × 2.1 mm (Phenomenex, Torrance, CA, USA) with the thermostat set at 40 °C. The mobile phase consisted of water (A) and acetonitrile (B) without additives. The gradient elution was carried out at a constant flow rate of 0.5 mL/min. The following gradients were applied: 0 min, 5% B; 2 min, 95% B; 4 min, 95% B; and 5 min, 5% B. A return to the initial gradient compositions (95% A and 5% B) was performed at 2 min. Total analysis time was 7 min.

Detection of 1,5-AG and IS was achieved using a triple-quadrupole mass spectrometer (LCMS-8050, Shimadzu, Kyoto, Japan). The spectrometer was equipped with an atmospheric-pressure chemical ionization (APCI) source; determination of the investigated substances was carried out in negative MRM mode. The following MS parameters were fixed: nebulizing gas flow, 3 L/min; heating gas flow, 10 L/min; interface temperature, 300 °C; desolvation line (DL) temperature, 200 °C; heat block temperature, 400 °C; and drying gas flow, 10 L/min. A summary of precursor and product ions, CEs, dwell time, Q1–Q3 pre-bias voltages, and retention times for each compound is presented in Table 2.

### 2.5. Method Development and Validation

#### 2.5.1. Glucose and Lactate

To determinate the intra- and inter-day precision and accuracy (*n* = 5), analyses were performed using two levels of multi control samples (PreciControl ClinChem Multi 1 and 2, cobas^®^, Roche Diagnostics GmBH, Mannheim, Germany). The target values were 103 and 243 mg/dL for glucose and 15 and 34.3 mg/dL for lactate. The calibrators were stored at 4 °C. Determinations of inter-day precision and accuracy were performed for five consecutive days. The results of intra- and inter-day validations are presented in Table 3.

#### 2.5.2. Acetone

The method used for determining acetone had been previously developed and validated in our department. In order to revalidate the method and check the quality of its operations, we decided to determine intra- (*n* = 5) and inter-day (*n* = 5) precision and accuracy for three control levels: low, 258 µmol/L; medium, 1075 µmol/L; and high, 8600 µmol/L. For validation, we used a Multicomponent Alcohol Calibrator Kit (Cerilliant, Round Rock, TX, USA). A 100 µg/mL calibrator was diluted with water (LC-MS grade) to a working solution of 15 µg/mL (258 µmol/L), while a 250 µg/mL calibrator was diluted to a working solution of 62.5 µg/mL (1075 µmol/L). In addition, we performed determinations using a 500 µg/mL (8600 µmol/L) calibrator. The solutions were stored at 4 °C. The results of intra- and inter-day validation are presented in Table 4.

#### 2.5.3. β-hydroxybutyric Acid

The method for the β-hydroxybutyric acid had been previously developed and validated in our department. The method was revalidated by preparing a methanol working solution of sodium DL-3-hydroxybutyrate (BHB) at the following concentration: 260, 1040, and 10,000 μg/mL. The stock solution and standard solutions were stored at −20 °C. Quality control samples (QC) were prepared by spiking the appropriate working solution into albumin standard: water (50:50, *v/v*) samples. The final concentrations of the QC samples were as follows: low, 26 μg/mL (250 µmol/L); medium, 104 μg/mL (1000 µmol/L); and high, 1000 μg/mL (9615 µmol/L). The intra- and inter-day precision and accuracy were determined in five repetitions. The results of the method validation are presented in Table 4.

#### 2.5.4. 1,5-anhydroglucitol

A method for determining 1,5-AG was implemented for the first time at our laboratory. The method turned out to be non-specific for determinations in urine and, therefore, validation was done for serum and whole blood. The working solutions of different concentrations were prepared by diluting standard solution with water (LC-MS grade). The stock solution and standard solutions were stored at −20 °C. Standard solutions were diluted with water to obtain working standard solutions at the following concentrations for 1,5-AG: 0.5, 1.25, 2.5, 5.0, 12.5, 50.0, 125.0, and 500.0 μg/mL. Calibration points and quality control (QC) samples were prepared by spiking the appropriate working solution into Biochemistry Control Serum level I samples (Biosystems, Barcelona, Spain), which were free from 1,5-AG, and into whole-blood samples. The final concentrations of the calibrators in serum were 0.1 (limit of detection; LOD), 0.25 (lower limit of quantification; LLOQ), 0.50, 1.0, 2.0, 5.0, 10.0, 20.0, and 50.0 (upper limit of quantification; ULOQ) μg/mL, while for blood they were 0.1 (LOD), 0.25, 0.50 (LLOQ), 1.0, 2.0, 5.0, 10.0, 20.0, and 50.0 (ULOQ) μg/mL. QC samples were prepared by spiking serum/blood to final concentrations of 0.5, 5.0, and 50.0 μg/mL for 1,5-AG.

Linearity was evaluated analyzing 1,5-anhydroglucitol working solutions with serum/human blood in final concentrations of 0.1, 0.25, 0.50, 1.0, 2.0, 5.0, 10.0, 20.0, and 50.0 µg/mL. The linearity of the calibration curve was determined by plotting the peak area ratio of 1,5-AG to IS in serum/human blood against the corresponding concentration ratio for assessment of method performance. The coefficient of determination (R^2^) and the calibration line equation were determined.

The intra- (*n* = 5) and inter-day (*n* = 5) precisions of the method were estimated by replicating analysis of QC samples. The precision was defined as relative standard deviation × 100% (SD/mean value × 100%).

The intra- (*n* = 5) and inter-day (*n* = 5) accuracies were expressed as mean relative error (MRE), where MRE% = (mean of the measured concentration − nominal concentration)/nominal concentration × 100%.

The recovery, matrix effect, and process efficiency were determined using the protocol of Matuszewski et al. [12]. The data are presented in Table 5.

### 2.6. Statistics

The statistical analyses were performed using the IBM SPSS Statistics 25 software package. A chi-squared test was used to check the equivalence of the compared groups as well as to examine the correlation between the nominal variables. A student’s *t*-test was used to check if there were statistically significant differences between the two independent groups. Spearman’s rank correlation coefficient was used to check whether there was a statistically significant correlation between the studied variables. Additionally, in the analysis of the results, the frequency (*n*, %) was analyzed, and the mean, standard deviation (SD), median, minimum, maximum, and lower and upper quartiles was analysed in the case of quantitative variables. A value of *p* < 0.05 was assumed as statistically significant.

## 3. Results

### 3.1. Glucose and Lactate

The mean serum glucose concentration in the control group was significantly higher compared to the study group. However, the mean glucose concentration in urine and VH was significantly higher in the study group. Detailed descriptive statistics for glucose levels are shown in Table 6.

No significant differences were found between the study and control groups in term of the mean lactate concentration for all three biological materials (serum, urine, VH). Detailed descriptive statistics for lactate levels are shown in Table 6.

### 3.2. Acetone

**Figure 1:**
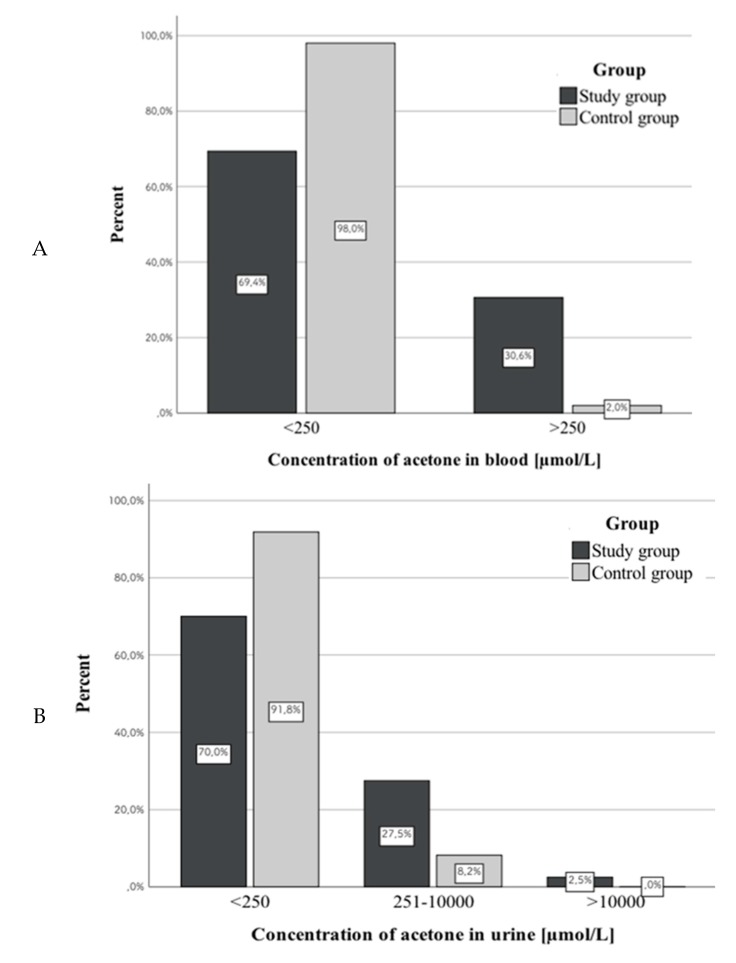

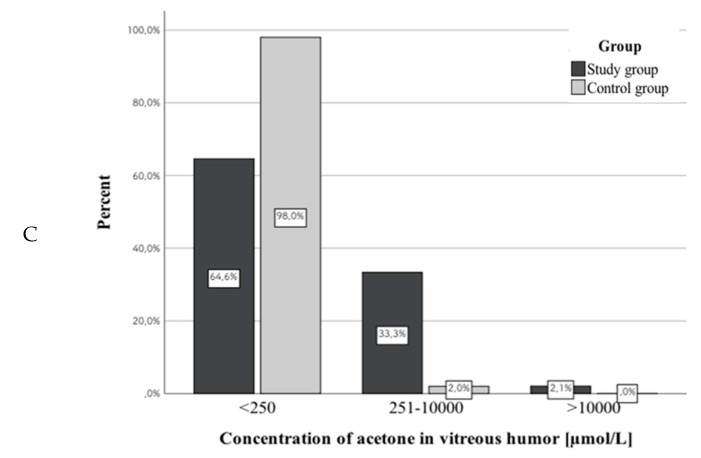
Correlations between the compared groups of subjects and acetone concentration in blood (**A**), urine (**B**), and vitreous humor (**C**).

Due to the fact that most of the results did not exceed LLOQ (250 µmol/L), we divided the results into three groups: ≤250, 251–10,000, and >10,000 µmol/L. According to this division, we observed statistically significant differences between the study and control group for all biological materials (whole blood, urine, VH): blood, λ^2^(1) = 14.59, *p* < 0.001 (study vs. control group); urine, λ^2^(2) = 7.39, *p* < 0.05 (study vs. control group); and VH, λ^2^(2) = 18.25, *p* < 0.001 (study vs. control group). Concentrations below 250 µmol/L were observed more often in control group (69.4% vs. 98.0% in blood, 70.0% vs. 91.8% in urine, and 64.6% vs. 98% in VH). In addition, urine and VH tests showed results >10,000 µmol/L in cases from the study group (2.5% in urine, 2.1% in VH). Figure 1 shows correlations between the compared groups of subjects and acetone concentrations in different biological materials.

### 3.3. β-hydroxybutyric Acid

**Figure 2:**
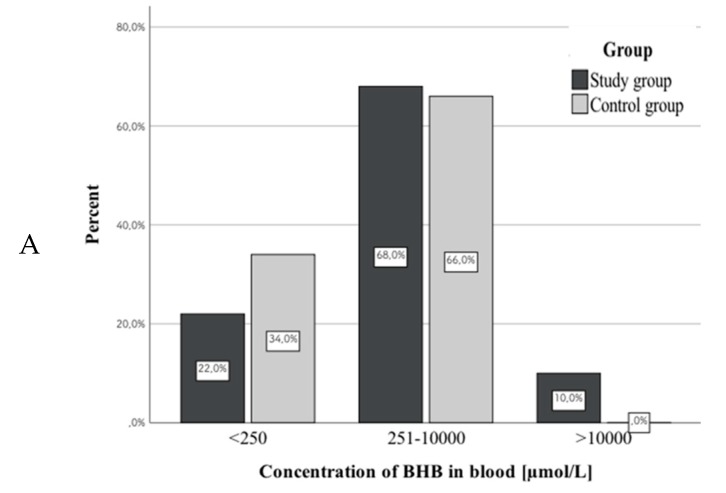

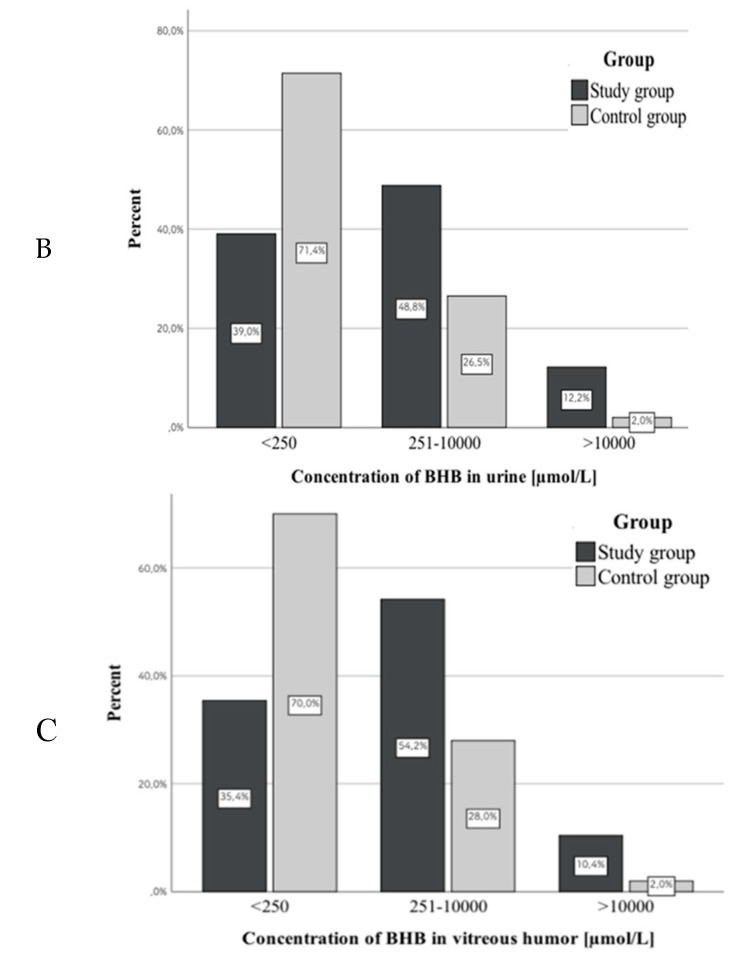
Correlations between the compared groups of subjects and β-hydroxybutyric acid (BHB) concentration in blood (**A**), urine (**B**), and vitreous humor (**C**).

As in the case of acetone, the results were divided into three groups. For all three biological materials (whole blood, urine, and VH), we found significant differences between study and control group: blood, λ^2^(2) = 6.3, *p* < 0.05 (study vs. control group); urine, λ^2^(2) = 10.6, *p* < 0.01 (study vs. control group); and VH, λ^2^(2) = 12.46, *p* < 0.01 (study vs. control group). Higher concentrations were observed more often in cases from the study group. In addition, for all three biological materials there were cases where concentrations exceeded 10,000 µmol/L; however, such cases occurred more often in the study group (10.0% vs. 0% in blood, 12.2% vs. 2.0% in urine, and 10.4% vs. 2.0% in VH). Figure 2 shows correlations between the compared groups of subjects and BHB concentrations in different biological materials. Table 7 presents the results of descriptive statistics for quantifiable results (within the range between 251–10,000 µmol/L). Considering these results, significant differences occurred between mean BHB concentrations in urine and VH.

### 3.4. 1,5-anhydroglucitol

**Figure 3:**
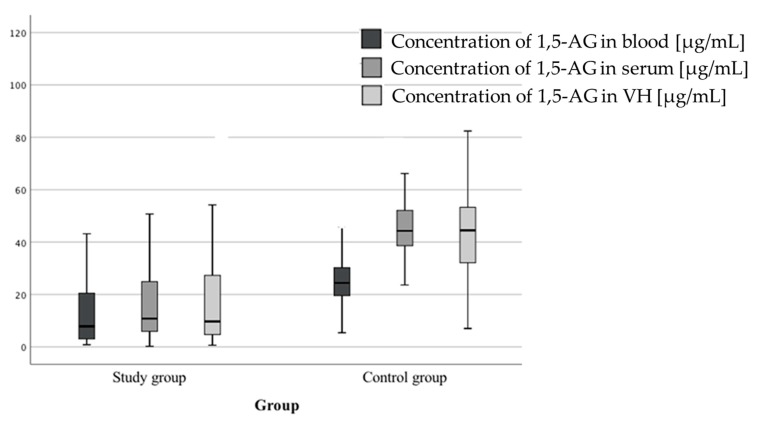
Comparison of 1,5-anhydroglucitol (1,5-AG) concentrations in different biological materials for both study and control groups.

In the case of 1,5-AG, for all tested biological matrices (serum, whole blood, vitreous humor), the mean concentration of this marker was significantly lower in the study group. Results of the analysis are presented in Table 8 and Figure 3.

### 3.5. PMI and Concentrations of Studied Markers

We also assessed whether there was a significant correlation between PMI (time from death to sampling) and the analyzed changes in the compared groups of subjects (Table 9). In the case of acetone determination, there were too few results within the linearity range of the method to make a statistical analysis. We observed the presence of three statistically significant correlations:

**Study group:**

The longer the PMI, the higher the lactate concentration in VH;

The longer the PMI, the lower the concentration of BHB in VH.

**Control group:**

The longer the PMI, the higher the concentration of 1,5-AG observed in VH.

### 3.6. Assessment of the Correlation between Concentrations of the Same Marker in Different Biological Matrices

#### 3.6.1. Glucose

In the study group, a significant correlation was observed between glucose concentration in serum and VH on the one hand, and in urine and VH on the other. In contrast, no significant correlations were observed in the control group (Table 10).

#### 3.6.2. Lactate

In the study group, we observed statistically significant correlations between lactate concentration in all biological materials (serum, urine, VH), while there was a significant correlation between serum concentration and VH in the control group (Table 11).

#### 3.6.3. β-hydroxybutyric Acid

In the study group, we observed a significant correlation between BHB concentration in blood and VH on the one hand, and in blood and urine on the other. In the control group, such a correlation was observed for the BHB concentration in blood and VH (Table 12).

#### 3.6.4. 1,5-anhydroglucitol

In the case of 1,5-AG, we observed the strongest correlation between the levels of this marker in different biological materials. In the study group, significant correlations occurred between concentrations in all studied biological materials (serum, whole blood, VH), and in the control group significant correlations were observed for concentrations in serum and whole blood (Table 13).

### 3.7. Assessment of the Correlation between Concentrations of the Analyzed Markers in Different Study Groups

We evaluated the concentrations of the analyzed short-term markers. We also considered the concentration of a long-term marker (glycated hemoglobin; HbA1c). Statistical analysis of correlations occurring in both groups in terms of HbA1c levels was discussed in detail in our previous study [10].

**Figure 4:**
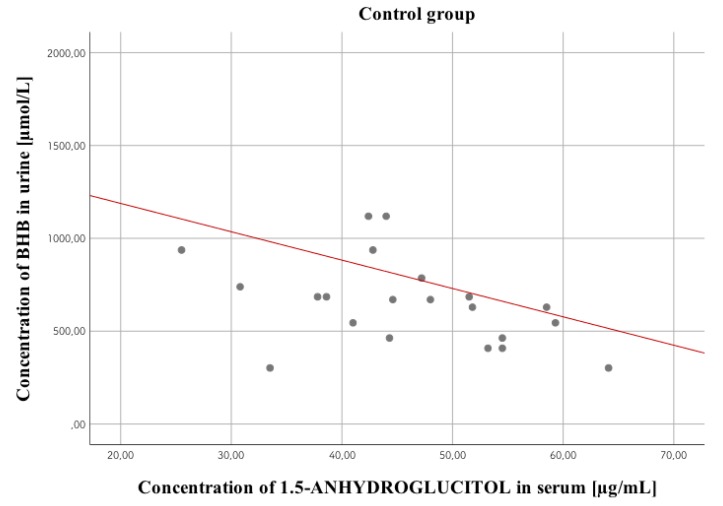
Correlation between serum 1,5-anhydroglucitol levels and urine BHB concentration in the control group. Dots correspond to the correlation between concentrations of both markers and the red line shows estimated correlation between markers.

Among the aforementioned correlations (Table 14), the strongest statistically significant positive correlation occurred in the study group between urine glucose concentration and HbA1c concentration, *r* = 0.48, *p* < 0.01, while the strongest statistically significant negative correlation occurred in control group between serum 1,5-AG concentration and urine BHB concentration, *r* = −0.5; *p* < 0.05 (Figure 4).

## 4. Discussion

Ante-mortem hyperglycemia (increased blood glucose levels) provides the basis for further diagnosis of diabetes or glycemic disorders. Tanatochemical processes call into question the usefulness of blood glucose determinations postmortem. There is a progressive postmortem decrease in blood glucose concentration in the interlethal period due to glucose consumption by body cells and bacteriae as well as an increase in lactate concentration resulting from postmortem anaerobic glycolysis [13].

In our research, the mean serum glucose concentration in the control group was significantly higher compared to the study group, while the mean glucose concentration was significantly higher in urine and VH for the study group. In addition, we observed intra-group variations of glucose concentrations, e.g., for urinary glucose in the study group, as the SD was 846 mg/dL. In this group most of results of urinary glucose were below 50 mg/mL, but some were high (over 1000 or 3000 mg/dL), which caused such differences. This may be partly due to the fact that some of the study group cases had well-controlled diabetes. Interferences in glucose determination may be another cause. This may indicate the low usefulness of this marker in postmortem determinations and confirms earlier reports [2,14,15]. However, we found many positive and negative correlations between glucose and lactate concentrations, as well as with other markers in both the study and control groups. Karlovsek [13] showed that glucose levels in VH >234 mg/dL or combined values of glucose and lactate in VH >427 mg/dL can be an indicator of the pre-mortem hyperglycemic state with a fatal outcome. Zilg et al. [16] performed glucose and lactate determinations in VH samples collected from the deceased as soon as possible after death and for a second time at the autopsy 1–3 days later. The researchers found that after an initial decrease in glucose concentration in VH in very early postmortem period, the concentration stayed stable for an appreciable time postmortem. They also suggest that glucose concentration in VH of 180 mg/dL (10 mmol/L) should be considered a marker of diabetic coma.

In the case of lactates, we were unable to show significant differences in the levels of this marker in both groups, which undermines the usefulness of this marker in postmortem diagnosis of deaths caused by hyperglycemia. In addition, we should mention the actual problems that we came across during determination of both glucose and lactates. For most test samples, especially serum, we had to perform a number of repetitions using dilutions of the material. The manufacturer of tests for glucose and lactate determinations declares no interference from total hemoglobin if its concentration in the test sample does not exceed 1000 mg/dL [17,18]. Serum collected postmortem is in most cases hemolyzed; consequently, the suggested value of total hemoglobin is exceeded, causing determination errors. Perhaps a more suitable and alternative method for determining these parameters in postmortem material is hydrogen proton magnetic resonance spectroscopy (^1^H-MRS) [19]. Another difficulty is the lack of control materials dedicated to determining both markers in VH, although according to some researchers, it is the matrix of choice to avoid postmortem blood glycolysis [20]. Madea [21] claims that for VH, we need to avoid analytical methods that have been validated for serum or urine; instead, we should use methods that have been optimized just for VH to obtain precise measurements. The above conclusions are in line with Blana et al. [22], who showed empirically that the measurement of lactate with analytical methods, calibrated for serum and urine, cannot be easily applied to VH. These researchers believe, however, that VH is beneficial for many analytes as the eye is in a physically protected environment and is less affected by microbial metabolism or autolysis than blood. Nevertheless, other authors [20] point out that VH glucose concentration tends to decrease in the early postmortem period. Vivero et al. [23] confirmed the usefulness of glucose determination in VH samples. It is worth noting, however, that in their study the mean PMI was 14.9 h. In our research, we observed that in the study group lactate levels in VH increased together with an increase in PMI. Keltanen et al. [24] concluded that PMI should be considered when interpreting lactate levels in postmortem samples. Considering that in our study the mean PMI was seven days for the study group and five days for the control group, this further undermines the possibility of using this marker in postmortem determinations.

Ketone bodies, which include acetone, BHB, and acetoacetic acid, are not specific markers in diagnosis of diabetic ketoacidosis (DKA) and hyperglycemia. The main cause of ketonemia is thought to be intracellular glucose deficiency, which can be caused by starvation, insulin deficiency, or excessive physical exertion. At the same time, there is an overproduction of acetyl-CoA generated from degradation of fatty acids. Increased concentration of acetyl-CoA may be the result of a disruption of the oxidation-reduction potential of cells and an excess of insulin antagonists, e.g., due to hypothermia or improper (high-fat) diet [25]. Therefore, when diagnosing DKA, other conditions should be excluded, such as lactic acidosis, starvation ketosis, hypothermia, metabolic acidosis with high anion gap in the course of poisoning (e.g., with methyl alcohol or ethylene glycol), or alcoholic ketoacidosis (AKA). When differentiating DKA from AKA, it should be remembered that ketosis associated with AKA or fasting is often a response of the body to lowered blood glucose levels (hypoglycemia). Due to postmortem progression of anaerobic glycolysis, low glucose concentration determined postmortem does not have to correspond with the hypoglycemic state at the time of death [2].

Our research showed that in both groups most acetone concentrations were within values considered physiological (did not exceed LLOQ, 250 µmol/L). In the study group, these cases represented 69% in whole blood, 70% in urine, and 65% in VH, while in the control group they represented 98%, 92%, and 98% respectively. For this reason, we could not make a statistical analysis and assess differences between the groups in terms of pathological concentrations of acetone.

Previous studies questioned the usefulness of acetone in diagnosis of ketoacidosis. Keltanen et al. [26] concluded that determination of acetone alone is not adequate for diagnosis of ketoacidosis, as opposed to BHB. This was confirmed by Palmiere, who thought that BHB is a better exponent of ketoacidosis than acetone [2]. Brinkmann et al. [27] suggest that interpretation of the results of acetone determinations must be done with a simultaneous analysis of the alcohol and medical history of the deceased, histopathological assessment of the characteristics of chronic alcoholism, and exclusion of other causes of metabolic disorders, such as diabetes. Teresiński et al. [25] showed that determination of elevated levels of acetone in analysis of biological material for the presence of ethyl alcohol or volatile substances can only be regarded as a screening test and excludes the possibility of differentiating exogenous acetone poisoning from ketosis. The aforementioned researchers suggest that full diagnosis requires BHB determination as well as interpretation of the results of determinations of BHB itself or the sum of ketone bodies.

The results of our study show that BHB levels in both groups more often exceeded 250 µmol/L in all tested biological materials compared to acetone. Considering the results within the linearity range of the method (251–10,000 µmol/L), on the one hand we did not find differences in the mean blood BHB levels between study and control group. On the other hand, such statistically significant differences between the groups were observed for urine and VH. Therefore, we agree with the previous reports on the importance of performing BHB determinations in VH. In a study by Heninger [28] of BHB in VH involving 1795 cases of death, the author showed that BHB concentration in VH >6000 µmol/L with simultaneous glucose levels in this material >200 mg/dL indicates the presence of DKA, while cases where glucose levels did not exceed 200 mg/dL may be the result of AKA. In addition, Heninger [28] proposed the following ranges for BHB determinations in VH: <400 µmol/L, normal; 410–1200 µmol/L, slightly elevated; 1210–2000 µmol/L, moderately elevated; 2000–6000 µmol/L, significantly elevated; and >6000 µmol/L, almost always life-threatening condition. Elliot et al. [29] conducted BHB determinations in biological materials collected from 350 deceased subjects. These researchers propose the following ranges with respect to BHB levels in blood, urine, and VH: 50 mg/L (480 µmol/L), physiological concentration; 51–249 mg/L (490–2390 µmol/L), elevated; and over 250 mg/L (2400 µmol/L), pathological condition [2,29]. Similarly, Palmiere [2] proposed the concentration of 2500 µmol/L in VH as a criterion for postmortem diagnosis of ketoacidosis. Iten and Meier [30], after analyzing BHB determinations in blood collected from 69 deceased from the control group, suggest recognizing BHB levels of <500 µmol/L as normal, the range of 500–2500 µmol/L as elevated, and over 2500 µmol/L as pathological. Due to the small number of deceased subjects in our study, we did not divide BHB levels into normal, elevated, and pathological. Considering the observed correlation between PMI and BHB concentration in VH in the study group, we recommend caution in interpreting the results, especially when the analysis is performed only on this material.

The most promising short-term marker of glycemia in postmortem diagnosis appears to be 1,5-AG. It belongs to the group of polyols, so called polyhydric alcohols derived from sugars (containing more than two hydroxyl groups) [31]. In recent years, determination of 1,5-AG concentration as a marker of postprandial hyperglycemia during pregnancy or before surgeries has been growing in popularity [32]. The primary source of 1,5-AG is food. The total pool of 1,5-AG in the body consists of a balance between its supply with food and excretion. There is virtually no metabolism of 1,5-AG in the body; instead, it is extracted by the kidneys where it is 99.9% reabsorbed via the SGLT-4 sodium-glucose transporter. Only after exceeding the so called renal threshold for glucose (approximately 180 mg/dL), glucose competitively suppresses 1,5-AG reuptake, which results in reduced serum concentration and loss with urine [32]. As a result of our research, we showed that mean 1,5-AG levels in all biological materials (blood, serum, VH) were significantly lower in the study group (of diabetics) compared to the control group. In addition, we demonstrated a high correlation between the concentration of this marker in different biological matrices (blood, serum, VH). Takata et al. [33] showed a strong correlation between 1,5-AG levels in VH and cerebrospinal fluid (CSF). We also observed that in the control group 1,5-AG concentration increased with the increase in time from death to sampling, which should, however, have no effect on determining hyperglycemia as cause of death. The importance of determining this marker in diagnosis of hyperglycemia is confirmed by Hess et al. [9,11]. In studies carried out on serum samples collected from 315 patients (116 non-diabetics and 199 diabetics) and blood samples of 58 deceased (27 non-diabetics and 31 diabetics), they showed significantly lower levels in patients with diabetes. Sydow et al. [34] performed determinations of 1,5-AG in VH and cerebrospinal fluid, and also observed lower levels of this marker in both matrices in the diabetic group, especially in the deceased due to diabetic coma. The aforementioned authors suggest determination of 1,5-AG in VH samples, especially in cases where it is impossible to secure whole blood for HbA1c determination. Similarly, Takata et al. [33] confirmed the usefulness of 1,5-AG determination in VH. In addition, Hess et al. [11] demonstrated postmortem metabolic stability of 1,5-AG.

In contrast to Sydow et al. [35], we concluded that our method is not specific enough for determining 1,5-anhydroglicitol in urine. Substances endogenously present in this matrix caused interference by distorting peaks (due to coelution) corresponding to both 1,5-AG and internal standard despite the fact the we used the MRM mode. Sydow et al. [35] also reported interferences in urine 1,5-AG determinations, but due to the modification of the method, they determined that it was specific enough for postmortem diagnosis in this matrix. They observed lower levels of this marker in the diabetic group compared to non-diabetics.

In the course of our study, we demonstrated the existence of a number of significant correlations between concentrations of markers in VH compared to other matrices, especially serum and blood. This confirms the usefulness of this material in postmortem chemical analysis and the possibility of its use if blood cannot be collected or at the initial stage of decomposition of the corpse. This is in line with previous reports on the usefulness of VH in postmortem diagnosis [2,13,16,28,33,34,36,37,38,39,40].

Heninger [28] claims that VH is easier to collect than blood and hemolysis is less of a problem. However, Felby et al. [41] observed lower BHB levels in VH compared to blood. According to the researchers, this is related to protein binding in blood and differences in dry weight of VH and whole blood. Gagajewskiet al. [42] showed a good correlation between vitreous BHB and blood BHB levels. In our research, we observed significant correlations between BHB levels in blood and VH.

## 5. Limitations

A clear limitation of our study was insufficient information on the deceased, e.g., lack of information on how diabetes was managed in these subjects. Intra-group variations in concentrations of markers were one of the major obstacles, due to the inability to match all cases within a group, e.g., well- and not well-controlled diabetes in study group. In addition, in some cases we did not have at our disposal a full set of biological materials. This was due to, among other things, bladder injuries or significant blood extravasation as a result of sudden death, e.g., in traffic accidents (cases from the control group), catheterization of a patient during stay in a medical facility prior to death, and prioritization of analyses commissioned by judicial authorities.

Another limitation is the relatively small size of the study and control groups, which translates to heterogeneity, e.g., in terms of age. Variations in the average time from the moment of death until autopsy hindered interpretation of the results of our tests.

## 6. Conclusions

In our opinion the most suitable short-term marker of glycemia in postmortem diagnosis is 1,5-AG. The high correlation between concentrations of this marker in different biological materials and the possibility of hemolysis of serum samples confirms that determinations can be successfully performed in both whole blood and vitreous humor samples. At the same time, we believe that diagnosis can be supplemented with determinations of acetone and BHB; however, it should be remembered that elevated levels of ketone bodies are not specific to diabetic ketoacidosis.

Interpretation of only the results of postmortem biochemical tests is insufficient to determine the cause of death due to carbohydrate metabolism disorders. In our opinion it must be correlated with, among other things, information on the circumstances of death, medical history of the deceased, testimonies of witnesses or family members, other results of toxicological and histopathological tests, and results of external and internal examination of the corpse. It should also confirm or exclude co-existence of another cause of death.

Due to the demonstrated correlation between concentrations of the tested markers and the time that elapsed between death and material collection, we recommend preforming autopsy as soon as possible in cases of death due to hyperglycemia.

## Figures and Tables

**Table 1:** Multiple reaction monitoring conditions used in the GC-MS analyses of β-hydroxybutyric acid (BHB) and internal standard derivates.

Compound	Transition (m/z)	CE (V)	Event Time (s)	Retention Time (min)
BHB derivate	275.0 > 159.2 * 275.0 > 147.2 233.0 > 147.1	9 21 12	0.075	9.385
BHB-*d*_4_ derivate	279.0 > 163.2 * 279.0 > 147.5 237.0 > 146.9	6 30 33	0.075	9.375

* Ions selected for quantitative analysis.

**Table 2:** Multiple reaction monitoring conditions used in the UHPLC/ESI-MS/MS analyses of 1,5-anhydroglucitol and internal standard.

Compound	Precursor Ion (m/z)	Product Ion [m/z]	Dwell Time (msec)	Q1 Pre-Bias (V)	Collision Energy (V)	Q3 Pre-Bias (V)	Retention Time (min)
1,5-Anhydro-D-glucitol	163.2	101.0 * 112.9 58.9	81.0	12 12 12	12 15 24	19 21 23	0.73
1,5-Anhydro-D-glucitol-^13^C_6_	169.2	105.1 * 118.1 61.0	81.0	17 12 17	13 16 22	17 16 22	0.72

* Ions selected for quantitative analysis.

**Table 3:** Validation parameters of the method for determining glucose and lactate. QC samples: PreciControl ClinChem Multi 1 (low concentration) and 2 (high concentration) (cobas^®^, Roche Diagnostics GmBH, Mannheim, Germany).

Marker	Concentration of QC	Intra-Day Precision (%)	Intra-Day Accuracy (%)	Inter-Day Precision (%)	Inter-Day Accuracy (%)
**Glucose**	103 mg/dL	1.0	0.1	1.0	1.7
	243 mg/dL	0.3	0.0	0.8	0.3
**Lactate**	15 mg/dL	0.3	1.9	1.7	0.9
	34.3 mg/dL	0.5	−1.8	1.0	−0.5

**Table 4:** Validation parameters of the methods for determining acetone and β-hydroxybutyric acid; * the calibration line equation concerns mg/mL, ** the calibration line equation concerns μg/mL.

Parameter	Acetone		β-hydroxybutyric Acid	
The linear concentration range (µmol/L)	250–10,000		250–10,000	
The coefficient of determination (R^2^)	0.9997		0.9968	
The calibration line equation	y = 4.7468x + 0 *		y = 0.0945x − 0.1593 **	
Intra-day precision (%)	258 µmol/L	5.2	250 µmol/L	2.7
	1075 µmol/L	5.9	1000 µmol/L	3.4
	8600 µmol/L	2.0	9615 µmol/L	4.0
Intra-day accuracy (%)	258 µmol/L	9.7	250 µmol/L	3.0
	1075 µmol/L	−2.3	1000 µmol/L	−3.1
	8600 µmol/L	3.1	9615 µmol/L	−3.1
Inter-day precision (%)	258 µmol/L	7.0	250 µmol/L	9.7
	1075 µmol/L	2.7	1000 µmol/L	5.4
	8600 µmol/L	2.7	9615 µmol/L	3.2
Inter-day accuracy (%)	258 µmol/L	9.7	250 µmol/L	−6.9
	1075 µmol/L	3.5	1000 µmol/L	−10.8
	8600 µmol/L	3.8	9615 µmol/L	−4.1

**Table 5:** Validation parameters of the method for determining 1,5-anhydroglucitol.

Parameter		Serum	Whole Blood
The linear concentration range (µg/mL)		0.25–50	0.50–50
LOD (limit of detection; µg/mL)		0.10	0.10
LLOQ (lower limit of quantification; µg/mL)		0.25	0.50
The coefficient of determination (R^2^)		0.9998	0.9999
The calibration line equation		y = 0.335x + 0	y = 0.0484x + 0.0464
Recovery (%)	0.5 µg/mL	93.1	90.4
	5.0 µg/mL	98.3	115.0
	50 µg/mL	108.3	103.3
Matrix effect (%)	0.5 µg/mL	102.4	112.8
	5.0 µg/mL	92.9	87.8
	50 µg/mL	93.4	86.2
Process efficiency (%)	0.5 µg/mL	95.3	102.0
	5.0 µg/mL	91.3	100.9
	50 µg/mL	101.2	89.1
Intra-day precision (%)	0.5 µg/mL	5.7	2.3
	5.0 µg/mL	4.1	4.2
	50 µg/mL	0.8	5.2
Intra-day accuracy (%)	0.5 µg/mL	−0.3	6.6
	5.0 µg/mL	9.1	6.9
	50 µg/mL	11.9	−0.6
Inter-day precision (%)	0.5 µg/mL	3.3	6.4
	5.0 µg/mL	7.0	3.8
	50 µg/mL	2.9	7.4
Inter-day accuracy (%)	0.5 µg/mL	−2.4	2.6
	5.0 µg/mL	8.2	5.8
	50 µg/mL	10.2	0.1

**Table 6:** Descriptive statistics for glucose and lactate determinations in the study and control groups.

Marker	Group		Descriptive Statistics					
			M	SD	Me	Lower Quartile	Upper Quartile	The Result of the Statistical Test
Glucose	Concentration in serum (mg/dL)	Study group	209	222	151	46	302	*t*(89.82) = 2.51; *p* < 0.05
		Control group	344	303	291	83	579.5	
	Concentration in urine (mg/dL)	Study group	382	846	21	9	226	*t*(41.59) = 2.28; *p* < 0.05
		Control group	65	203	13	6.5	23.5	
	Concentration in vitreous humor (mg/dL)	Study group	119	216	9	5	154	*t*(50.45) = 2.98; *p* < 0.05
		Control group	23	48	7	4.5	14	
Lactate	Concentration in serum (mg/dL)	Study group	374	115	360	306	461	*t*(96) = 0.89; *p* > 0.05
		Control group	420	121	437.5	348	517	
	Concentration in urine (mg/dL)	Study group	208	138	208.5	72	310	*t*(91) = 0.19; *p* > 0.05
		Control group	207	202	148.5	98	253.5	
	Concentration in vitreous humor (mg/dL)	Study group	359	137	357	282	460	*t*(96) = 0.15; *p* > 0.05
		Control group	314	124	304	221.5	409	

M, mean; SD, Standard deviation; Me, median.

**Table 7:** Descriptive statistics for BHB determinations in the study and control groups for results in the range from 251 to 10,000 µmol/L.

Group		Descriptive Statistics					
		M	SD	Me	Lower Quartile	Upper Quartile	The Result of the Statistical Test
Concentration in whole blood	Study group	1260	1602	582	414	1042	*t*(90) = 1.29; *p* > 0.05
	Control group	850	1436	352	297.5	936	
Concentration in urine	Study group	1970	2161	1658	415	2253	*t*(35.15) = 2.76; *p* < 0.01
	Control group	792	688	670	463	824	
Concentration in vitreous humor	Study group	1752	2327	868	419	1590	*t*(50.63) = 2.23; *p* < 0.05
	Control group	826	837	565	275	1274	

**Table 8:** Descriptive statistics for 1,5-anhydroglucitol determinations in the study and control groups.

Group		Descriptive Statistics					
		M	SD	Me	Lower Quartile	Upper Quartile	The Result of the Statistical Test
Concentration in whole blood	Study group	12.7	12.3	7.8	2.9	20.8	*t*(98) = 5.61; *p* < 0.001
	Control group	26.5	12.3	24.2	18.7	31.8	
Concentration in serum	Study group	18.6	17.8	12.4	5.9	26.7	*t*(80.92) = 8.78; *p* < 0.001
	Control group	45.4	11.7	44.2	33.8	52.2	
Concentration in vitreous humor	Study group	17.5	16.9	9.1	4.7	27.3	*t*(94) = 7.83; *p* < 0.001
	Control group	43.3	15.4	44.5	31.6	53.3	

**Table 9:** Correlation between PMI (time from death to collection of biological material) and the concentrations of the analyzed markers. Significant correlations are in bold.

Marker	PMI					
	Study Group			Control Group		
	Serum	Urine	Vitreous Humor	Serum	Urine	Vitreous Humor
Glucose	−0.18	0.002	0.18	0.06	0.01	0.14
Lactate	0.15	0.23	**0.45**	−0.09	0.02	0.13
1,5-anhydroglucitol	−0.16 (blood)	−0.05 (serum)	−0.06	0.18 (blood)	0.04 (serum)	**0.29**
BHB	−0.27	−0.31	**−0.44**	0.15	0.21	0.01

**Table 10:** Correlation between glucose concentration in different biological materials. Significant correlations are in bold.

Glucose				
Biological Material		Serum	Urine	Vitreous Humor
Serum	Study group	-	0.25	**0.39**
	Control group	-	0.04	−0.09
Urine	Study group	0.25	-	**0.61**
	Control group	0.04	-	0.26
Vitreous humor	Study group	**0.39**	**0.61**	-
	Control group	−0.09	0.26	-

**Table 11:** Correlation between lactate concentration in different biological materials. Significant correlations are in bold.

Lactate				
Biological Material		Serum	Urine	Vitreous Humor
Serum	Study group	-	**0.4**	**0.61**
	Control group	-	−0.07	**0.54**
Urine	Study group	**0.4**	-	**0.51**
	Control group	−0.07	-	0.04
Vitreous humor	Study group	**0.61**	**0.51**	-
	Control group	**0.54**	0.04	-

**Table 12:** Correlation between BHB concentration in different biological materials. Significant correlations are in bold.

β-hydroxybutyric Acid				
Biological Material		Blood	Urine	Vitreous Humor
Blood	Study group	-	**0.46**	**0.77**
	Control group	-	0.002	**0.63**
Urine	Study group	**0.46**	-	0.33
	Control group	0.001	-	0.4
Vitreous humor	Study group	**0.77**	0.33	-
	Control group	**0.63**	0.41	-

**Table 13:** Correlation between 1,5-anhydroglucitol concentration in different biological materials. Significant correlations are in bold.

1,5-anhydroglucitol				
Biological Material		Blood	Serum	Vitreous Humor
Blood	Study group	-	**0.77**	**0.66**
	Control group	-	**0.59**	**0.56**
Serum	Study group	**0.77**	-	**0.7**
	Control group	**0.59**	-	**0.81**
Vitreous humor	Study group	**0.66**	**0.7**	-
	Control group	**0.56**	**0.81**	-

**Table 14:** Assessment of the correlation between concentrations of the analyzed markers in different study groups. Positive correlation: as the concentration of one marker increases, so does the concentration of the second marker; negative correlation: as the concentration of one marker increases, the concentration of the second marker decreases. HbA1c – glycated haemoglobin.

	Study Group	Control Group
**Positive Correlation**	Serum glucose concentration versus serum lactate concentration, *r* = 0.31; *p* < 0.05 Urine glucose concentration versus HbA1c levels, *r* = 0.48; *p* < 0.01 VH glucose concentration versus VH lactate levels, *r* = 0.3; *p* < 0.05 VH glucose concentration versus HbA1c levels, *r* = 0.38; *p* < 0.05 Serum lactate concentration versus HbA1c levels, *r* = 0.34; *p* < 0.05	Serum glucose concentration versus serum lactate concentration, *r* = 0.31; *p* < 0.05 Urine glucose concentration versus urine lactate concentration, *r* = 0.32; *p* < 0.05
**Negative Correlation**	VH 1,5-AG concentration versus HbA1c concentration, *r* = –0.38; *p* < 0.01 VH glucose concentration versus VH 1,5-AG concentration, *r* = –0.38; *p* < 0.01	VH glucose concentration versus VH 1,5-AG concentration, *r* = –0.3; *p* < 0.05 Urine glucose concentration versus VH 1,5-AG concentration, *r* = –0.31; *p* < 0.05 Urine lactate concentration versus VH 1,5-AG concentration, *r* = –0.35; *p* < 0.05 VH lactate concentration versus VH 1,5-AG concentration, *r* = –0.31; *p* < 0.05 Serum 1,5-AG concentration versus urine BHB concentration, *r* = –0.5; *p* < 0.05
